# The Social Life of *Aeromonas* through Biofilm and Quorum Sensing Systems

**DOI:** 10.3389/fmicb.2017.00037

**Published:** 2017-01-20

**Authors:** Emilie Talagrand-Reboul, Estelle Jumas-Bilak, Brigitte Lamy

**Affiliations:** ^1^Équipe Pathogènes Hydriques Santé Environnements, UMR 5569 HSM, Université de MontpellierMontpellier, France; ^2^Département d'Hygiène Hospitalière, Centre Hospitalier Régional Universitaire (CHRU) de MontpellierMontpellier, France; ^3^Département de Bactériologie, Centre Hospitalier Universitaire (CHU) de NiceNice, France

**Keywords:** biofilm, quorum sensing, multicellularity, coordination, cooperation, social life, bacterial communities, virulence

## Abstract

Bacteria of the genus *Aeromonas* display multicellular behaviors herein referred to as “social life”. Since the 1990s, interest has grown in cell-to-cell communication through quorum sensing signals and biofilm formation. As they are interconnected, these two self-organizing systems deserve to be considered together for a fresh perspective on the natural history and lifestyles of aeromonads. In this review, we focus on the multicellular behaviors of *Aeromonas*, i.e., its social life. First, we review and discuss the available knowledge at the molecular and cellular levels for biofilm and quorum sensing. We then discuss the complex, subtle, and nested interconnections between the two systems. Finally, we focus on the aeromonad multicellular coordinated behaviors involved in heterotrophy and virulence that represent technological opportunities and applied research challenges.

## Introduction

The practices of clinical bacteriology and research in microbiology have long been subjected to the principle of “pure strains”, which limited analysis to unicellular/monoclonal organisms. However, the multicellular/polyclonal lifestyle becomes increasingly important for understanding bacteria, as strengthened by the sociomicrobiology aspects of biofilm formation and quorum sensing (Parsek and Greenberg, 2005; Claessen et al., 2014). These biological mechanisms are particularly studied in the environmental opportunistic pathogen, *Pseudomonas aeruginosa* and have been confirmed as major virulence factors explaining aspects of *P. aeruginosa* pathogenesis, mainly in cystic fibrosis and health-care associated infections (Bjarnsholt et al., 2010). Rather than true virulence factors, biofilm formation and quorum sensing are adaptive traits involved in the versatile lifestyle of *P. aeruginosa* in natural ecosystems, which become patho-adaptive in human and health-care ecosystems.

Aeromonads represent another interesting group of bacteria for such multicellular functioning. The genus *Aeromonas* belongs to *Aeromonadaceae* family, *Aeromonadales* order and *Gammaproteobacteria* class. These bacteria are gram-negative, facultative anaerobic, oxidase and catalase positive, fermentative, and mostly motile bacilli. Aeromonads are common inhabitants of aquatic environments such as fresh, estuarine, marine waters, and sediments and are found in association with animals. *Aeromonas* are environmental opportunistic pathogens of animals and human. Aeromonads are responsible for furunculosis and septicemia in fish. In human, they can cause gastroenteritidis, wound infections, bacteraemia, and less frequently respiratory infections, hepatobiliary infections, peritonitis, urinary tract infections, and ocular infections (Janda and Abbott, 2010). Among the 30 species recognized to date in this genus, the most studied are *A. dhakensis, A. hydrophila, A. caviae, A. veronii*, and *A. salmonicida*, which correspond to relevant species for human and animal infections. The members of *Aeromonas* are characterized by a remarkably ability to colonize a wide range of habitats. Typically, many of its colonization aspects rely on biofilm production and cell-cell signaling. Numerous studies have been conducted on these two aspects, and a large amount of data is available but mostly scattered in the literature. These data have never been collected into an integrative perspective of community dynamics.

In this review, we focus on the multicellular behavior of *Aeromonas*, referred to here as “social life.” First, we review and discuss the available knowledge at the molecular and cellular levels for biofilm and quorum sensing. We then discuss the complex, subtle, and nested interconnections between the two systems and highlight the current gaps in knowledge. Finally, we focus on aeromonad multicellular coordinated behaviors involved in heterotrophy and virulence that represent applied research challenges and technological opportunities.

## Biofilm formation in *Aeromonas*

**Figure 1:**
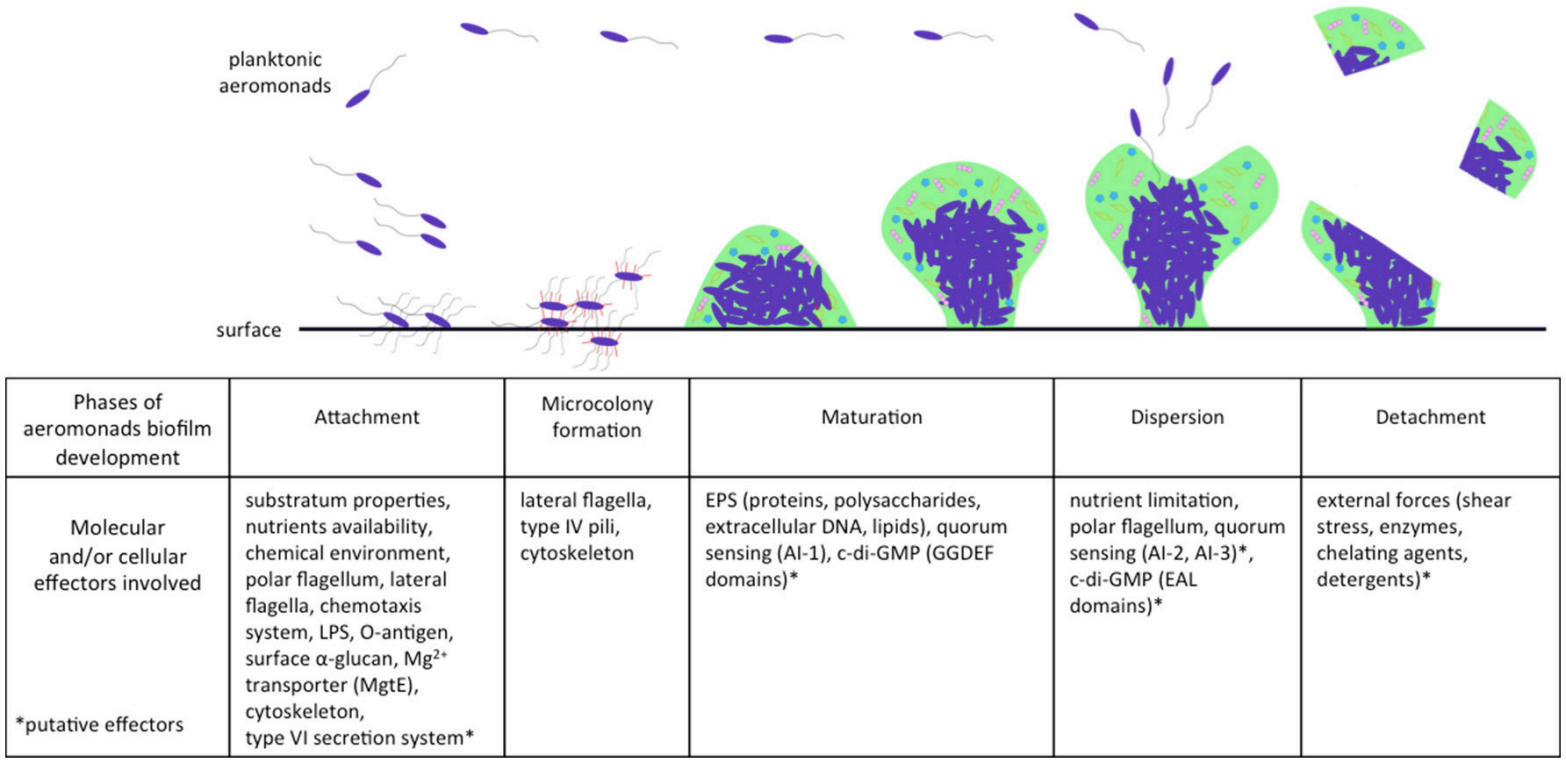
**Effectors involved in different phases of biofilm development in aeromonads**. Planktonic aeromonads initiate the formation of biofilm on surface under influence of environmental conditions. Several bacterial factors are involved in the attachment step, including flagella and other external structures, chemotaxis system, and cytoskeleton. After division, bacteria that were well-aggregated, attached to the surface to form a microcolony. Biofilm acquires its mechanical stability by the production of an EPS matrix encompassing proteins, polysaccharides, extracellular DNA, and lipids. The AI-1 quorum sensing system enhances the maturation of biofilm, which is likely related to the second messenger c-di-GMP involved in the bacterial transition from planktonic to sessile lifestyle. When the conditions of life in biofilm deteriorate (e.g., nutrient limitation), a dispersion phase occurs and aeromonads escape from biofilm and return to the planktonic lifestyle. In another case, the biofilm can be detached by external stress (e.g., shear forces). AI-1, Autoinducer-1 quorum sensing system; AI-2, Autoinducer-2 quorum sensing system; AI-3, Autoinducer-3 quorum sensing system; EAL, protein domains harboring phosphodiesterase activity involved in the c-di-GMP degradation; EPS, extracellular polymeric substances; GGDEF, protein domains harboring guanylate synthase activity involved in the c-di-GMP synthesis; LPS, lipopolysaccharides.

A majority of bacteria, including aeromonads, live attached to biofilms on biotic or abiotic surfaces. Natural biofilms are developed and differentiate themselves to build a packed community that is often multi-species and is embedded in a polymeric extracellular matrix of their own production. This matrix contains channels that are included for the circulation of nutrients and water (Donlan and Costerton, 2002). The architecture of bacterial biofilms has largely been documented as confocal scanning laser microscopes have come into use (Donlan and Costerton, 2002). Like *P. aeruginosa* (Sauer et al., 2002; Klausen et al., 2006), the natural history of biofilm formation in aeromonads includes the classical steps of attachment, microcolony formation, maturation, and dispersion (Figure 1).

### Attachment and promoting factors

This first step, attachment, is pivotal for biofilm formation (Figure 1). Aeromonads are able to colonize both biotic surfaces in plants and animals (Mizan et al., 2015), and abiotic surfaces, notably sediment, steel, glass, and polyvinyl chloride (Zalmum et al., 1998; Béchet and Blondeau, 2003; Bomo et al., 2004; Doğruöz et al., 2009; Balasubramanian et al., 2012). The substratum properties, chemical components, and nutrient availability are critical conditions influencing bacterial attachment. For instance, Jahid et al. (2013, 2015) have shown that low salinity (0.25% wt./vol.) enhances biofilm formation by *Aeromonas hydrophila*, whereas glucose concentration above 0.05% (wt./vol.) impairs its formation. In addition, *Aeromonas* spp. harbor several structures and/or mechanisms, including flagella and chemotaxis, lipopolysaccharides (LPS), and other surface polysaccharides (α-glucan), Mg^2+^ transporters and cytoskeletons that are actively involved in the first steps of biofilm formation (Figure 1).

Motility is decisive for attachment, and any system that promotes motility may stimulate attachment. Among these systems, the constitutive polar flagellum of *Aeromonas* spp., responsible for swimming in liquid, plays a critical role in biofilm formation and contributes to colonization of surfaces, as demonstrated for *Aeromonas caviae* strain Sch3 and *A. hydrophila* {*A. piscicola*} strain AH-3 (Kirov et al., 2004; Merino et al., 2014). In *A. hydrophila*, the O-glycosylation of polar flagella seems to be a prerequisite for adhesion and biofilm formation because mutants with reduced flagella glycosylation are unable to form biofilms (Merino et al., 2014; Fulton et al., 2015). In addition to polar flagella, members of *Aeromonas* spp. display inducible lateral flagella distributed randomly on the cell surface (Kirov et al., 2002). These lateral flagella are responsible for the swarming motility, enabling bacteria to migrate over surfaces by rotative movements and the formation of side-by-side cell groups called rafts (Gavín et al., 2002; Kirov et al., 2002). They also contribute to biofilm formation for Aeromonads (Gavín et al., 2002, 2003). Similarly, swimming, swarming, and twitching motility are known to be pivotal for *P. aeruginosa* biofilm formation (Barken et al., 2008), but *Aeromonas* strains do not develop any detectable twitching motility (Kirov et al., 1999). Chemotaxis systems mediated by the histidine kinase CheA allow bacterial cells to navigate in chemical gradients by regulating bacterial flagellar motility and are necessary for swimming, swarming, and for biofilm formation (Porter et al., 2011). Consistent with this, a chemotactic mutant strain Δ*cheA* of *A. caviae* is unable to swim or swarm in agar assays, and its biofilm formation ability is decreased by more than 80% (Kirov et al., 2004). Ten clusters of chemotaxis genes have been described in the genome of *A. hydrophila* ATCC 7966^T^, including two gene-system homologs to the gene clusters I and V of *P. aeruginosa* PAO1 (Wuichet and Zhulin, 2010) shown to be essential for chemotactic motility (Masduki et al., 1995). The VgrG proteins corresponding to both components and effectors of the type VI secretion system (T6SS) of the *A. hydrophila* {*A. dhakensis*} strain SSU promote biofilm formation. Their function is not fully characterized but may occur at the attachment step because VgrG3 also enhances swimming motility (Sha et al., 2013).

The O-antigen is the most surface-exposed moiety of LPS, which acts as an attachment factor and enhances the formation of biofilm in *A. hydrophila* strains (Merino et al., 2014; Fulton et al., 2015). The cell surface hydrophobicity and charge conferred by LPS were involved in *P. aeruginosa* biofilm formation (Ruhal et al., 2015), but their exact roles in the *Aeromonas* biofilm development are not fully understood. The *A. hydrophila* surface α-glucan independent of the LPS also improves biofilm formation (Merino et al., 2012). In addition, Mg^2+^ is suspected to contribute to the integrity and stability of the outer membrane by preventing electrostatic repulsion between LPS molecules (Hancock, 1984). This divalent cation and its transporter MgtE are directly involved in adherence to epithelial cells, in swarming and in biofilm formation of *A. hydrophila* {*A. piscicola*} AH-3 (Merino et al., 2001). Genomic data confirm the links between MgtE and cell motility because the gene *mgtE* is adjacent to the polar flagellar operon *flg* in the strain *A. hydrophila* ATCC 7966^T^ (Seshadri et al., 2006). However, the level of evidence is still low for understanding how MgtE enhances biofilm formation. Finally, the protein MinD, a cytoskeletal ATPase involved in the septal placement of bacteria and plastid division sites in many bacteria (Shih and Rothfield, 2006), is also markedly involved in adherence, bacterial motility, and formation of biofilm in the *A. hydrophila* strain W (Huang et al., 2015).

### Microcolony formation and maturation

Once attachment is completed, cell division processes maintain bacterium to bacterium bonds and lead to microcolony formation (Figure 1; Lynch et al., 2002). Type IV pili and fimbriae participate in the formation of microcolonies and biofilm for strains of *Aeromonas* (Béchet and Blondeau, 2003; Kozlova et al., 2008). Three families of type IV pili structures (Bfp, Flp, and Tap) have been involved in microcolony and biofilm formation for several species, e.g., *Aggregibacter actinomycetemcomitans* and *Vibrio vulnificus* (Paranjpye and Strom, 2005; Perez-Cheeks et al., 2012). Bacteria of the genus *Aeromonas* also harbor the three families of type IV pili structures (Kirov et al., 1999; Boyd et al., 2008). Bfp has been shown to be critical for biofilm formation in *Aeromonas veronii* (Hadi et al., 2012), but the involvement of Flp and Tap has not yet been demonstrated in aeromonads. Further evaluation deserves to be conducted to more precisely specify the role of each type of pili in early steps of *Aeromonas* biofilm formation.

From microcolonies, colonies grow and the biofilm matures. After 48 h in a stainless steel flow-through model, Lynch et al. (2002) observed mushroom-like “large microcolonies” of *A. hydrophila*, characteristic of mature biofilms (Figure 1). Aeromonads live embedded in a self-produced matrix of extracellular polymeric substances (EPS), mainly composed of polysaccharides, proteins, nucleic acids, and lipids (Andersson et al., 2011). The resulting structure provides the mechanical stability of biofilms (Peterson et al., 2015). When forming biofilms, *A. hydrophila* produces more capsular and colloidal EPS compared to planktonic cells (Castro et al., 2014). As in other bacteria including *P. aeruginosa* (Rasamiravaka et al., 2015), the persistence of a mature biofilm in *Aeromonas* is largely controlled by signaling systems triggered by high bacterial density (Lynch et al., 2002; Khajanchi et al., 2009). These aspects are developed in the section “How are biofilm and quorum sensing interconnected?”

### Detachment and dispersion

Detachment corresponds to passive escape from a biofilm, occurring under the influence of external factors such as shear stress or degradation of extracellular polymeric matrix by enzymes, chelating agents, or detergents. In contrast, dispersion is an active mechanism of biofilm escape depending on biofilm growth, cell density, and related factors (Figure 1; Petrova and Sauer, 2016). Very few data on *Aeromonas* biofilm detachment and dispersion are available, and most knowledge relies on data from *P. aeruginosa* biofilm models. Dispersion is triggered by exogenous factors such as nutrient availability and toxic compounds, and by internal regulatory systems including quorum sensing systems (Kim and Lee, 2016). To enhance its dispersion, it is suspected that aeromonads degrade some compounds of their own extracellular polymeric matrix or other bacteria. Consistent with this, Bansal et al. (2015) showed that the depolymerase produced by an *Aeromonas punctata* strain is able to degrade the capsular polysaccharides of *Klebsiella pneumoniae* within a biofilm.

### Functions of biofilms

#### Biofilm acts as niche and reservoir

Biofilm formation is an emblematic example of niche construction, a process by which an organism alters its own environment in order to increase its chances of survival (Odling-Smee et al., 2003). Indeed, biofilms enhance stability and protect bacteria against external factors (Costerton et al., 1987; Peterson et al., 2015). First, bacterial sessile life is associated with an increased persistence and resistance to stressful conditions including salinity, antimicrobial substances, or oxidative stress, compared to the planktonic lifestyle (Van Acker et al., 2014). Second, biofilms provide cell nutrients in higher concentrations than the surrounding environment via the nutrient-rich solute retained in the interstitial region of the extracellular polymeric matrix (Tsuchiya et al., 2009, 2016).

The formation of biofilm is highly beneficial even if *Aeromonas* spp. are able to grow and live freely in water. Biofilms act as reservoirs in which some aeromonads are able to persist for several years and emerge later in favorable conditions (Kühn et al., 1997). Only certain clones seem to be able to persist; Villari et al. (2003) found that only two clones of *A. hydrophila* and *A. caviae* persisted in natural mineral freshwater over a 3-year study, while molecular heterogeneity was much higher in samples from stream waters running near the spring.

Aeromonads within a biofilm are more resistant to disinfectants than planktonic cells, as shown for *A. hydrophila* strains (Jahid and Ha, 2014). Aeromonads have thus been recovered from biofilm in drinking-water distribution systems (Chauret et al., 2001; September et al., 2007), even when water supply is chlorinated (Fernández et al., 2000).

#### Biofilm promotes gene exchange and antibiotic resistance

Biofilm structure provides a close cell-to-cell proximity that enhances genetic transfers, mainly conjugation and natural transformation (Hausner and Wuertz, 1999; Hendrickx et al., 2003; Madsen et al., 2012). The two types of biofilm-associated horizontal genetic transfers (HGT) have been demonstrated in the genus *Aeromonas* (Rhodes et al., 2000; Huddleston et al., 2013), but the transfer by phage transduction has not yet been observed within aeromonads biofilm.

In the genus *Aeromonas*, conjugation has even been demonstrated in experiments with aeromonads as donor cells and *E. coli* as recipient cells (Rhodes et al., 2000; Schmidt et al., 2001; Casas et al., 2005). *Aeromonas* can harbor the machinery for the type IV secretion system (T4SS), enabling the genetic conjugative transfer of mobile genetic elements between bacteria (Rangrez et al., 2006). Within biofilms, high cell density may facilitate conjugation between two aeromonads or between aeromonads and other bacteria.

Additionally, Huddleston et al. (2013) have demonstrated, using direct experimental assays, that most members of aeromonads are naturally competent for transformation (73% of 37 tested strains). The type IV pili of *Aeromonas* may enable the recipient cell to incorporate extracellular DNA. The analysis of “transformability” and “donatability” between *Aeromonas* strains showed that transformation of groups was constrained to phylogroups (Huddleston et al., 2013), consistent with some population studies that have highlighted HGT between close relatives in the genus *Aeromonas* (Silver et al., 2011; Roger et al., 2012b). The high concentration of extracellular DNA within a biofilm may facilitate the transformation of *Aeromonas*, as in other bacteria capable of natural transformation (Merod and Wuertz, 2014).

Evidence is available for HGT of 16S rDNA and housekeeping genes over the evolutionary history of the *Aeromonas* genus (Roger et al., 2012a,b). Moreover, mobile genetic elements are frequently recovered from aeromonad genomes, e.g., plasmids, transposons, insertion sequences, and integron-associated gene cassettes, as recently reviewed (Piotrowska and Popowska, 2015). These HGT are important in the evolution and fitness of this genus and may be enhanced in biofilm, as in other bacteria (Madsen et al., 2012).

Mobile genetic elements recovered from *Aeromonas* strains carry genes involved in virulence (T4SS, T6SS, T3SS compounds, and effectors), stress response (HipAB toxin/antitoxin system) and resistance to heavy metals (mercury) and toxic compounds (quaternary ammoniums), but the most frequently reported elements are antibiotic resistance genes (Piotrowska and Popowska, 2015).

In *Aeromonas*, acquired resistance increases the level of antibiotic resistance in both environmental and clinical strains (Esteve et al., 2015). The genetic support of these acquired resistances is transferable by chromosomal transposons/integrons or plasmids that carry genes associated with resistance to beta-lactamines, quinolones, macrolides, tetracycline, sulfonamides, and chloramphenicol (Janda and Abbott, 2010; Piotrowska and Popowska, 2015). *In vivo* transfer of TEM-24 plasmid-borne extended-spectrum β-lactamase, likely from human microbiota, was reported from enterobacteria to *Aeromonas* (Marchandin et al., 2003). Such antibiotic-resistance gene transfer has not yet been demonstrated within aeromonad biofilms, but it was reported for *K. pneumoniae* (Hennequin et al., 2012). The co-localization of resistance genes in mobile genetic elements can lead to cross-resistance to multiple families of antibiotics in aeromonads (Maravić et al., 2013).

Consequently, there are some concerns about the spread of resistance genes by transfer events within microbial communities, including aeromonads in surface biofilms, both in natural environments and care units.

### Mixed biofilms

Under environmental conditions, biofilms often mix several bacterial species including *Aeromonas* sp., as reviewed in Table 1. Aeromonads have been co-isolated with one or more representatives of other genera from natural freshwater and seawater biofilms (Stine et al., 2003; September et al., 2007; Balasubramanian et al., 2012; Farkas et al., 2012; Marti et al., 2014). *Aeromonas* spp. are also observed in mixed-species biofilms inside dental care plastic lines (Tall et al., 1995) or the surfaces of food plants (Gunduz and Tuncel, 2006).

**Table 1:** **Species of ***Aeromonas*** and co-isolated bacterial species from mixed biofilms**.

**Type of mixed biofilm**	***Aeromonas*** **species**	**Co-isolated species**	**References**
Lines of dental air-water syringes	*Aeromonas* sp.	*Pseudomonas* sp., *Pasteurella* sp., *Moraxella* sp. *Ochrobactrum* sp.	Tall et al., 1995
Coastal waters	*A. jandaei, A. trota, A. salmonicida*	*Shewanella baltica, Shewanella frigidmarina, Escherichia coli, Vibrio vulnificus*	Stine et al., 2003
Textile dye-degrading immobilized cell bioreactor	*Aeromonas* sp.	*Bacillus sp., Alcaligenes sp*.	Sharma et al., 2004
Ice-cream plant	*Aeromonas* sp.	*Enterobacter* sp., *Citrobacter* sp., *Leuconostoc/Pediococcus* sp., *Streptococcus* sp.,	Gunduz and Tuncel, 2006
Drinking water distribution system	*Aeromonas* sp.	*Pseudomonas* sp., *Klebsiella* sp., *Pantoea* sp., *Acinetobacter* sp.	September et al., 2007
Wastewater treatment process	*A. hydrophila*	*Comamonas testosteroni*	Li et al., 2008
Drinking water distribution system	*A. hydrophila*	*Pseudomonas aeruginosa, Enterococcus* sp., *Escherichia coli, Clostridium perfringens*	Farkas et al., 2012
Polyvinylchloride immersed in seawater	*Aeromonas* sp.	*Pseudomonas* sp., *Enterobacter* sp., *Flavobacterium* sp., *Cytophaga* sp., *Bacillus* sp., *Micrococcus* sp.	Balasubramanian et al., 2012
Chitin embedded into agarose beads	*A. hydrophila*	*Flavobacterium* sp.	Jagmann et al., 2012
Coaggregation and mixed-biofilm assays	*Aeromonas* sp.	*Bacillus cereus*	Cheng et al., 2014
Stone downstream of a wastewater discharge	*A. caviae*	*Klebsiella oxytoca*	Marti et al., 2014

Bacterial biofilms harbor some inherent degree of structural heterogeneity in response to chemical gradients and adaptation to local microenvironments. Heterogeneity also concerns the mixture of bacterial species in a biofilm; juxtaposition of bacteria occurs such that mutualistic interactions are facilitated (Stewart and Franklin, 2008). Experimentally, the *A. hydrophila* strain AH-1 and *Flavobacterium* sp. form a mixed biofilm on chitin-containing particles. The *A. hydrophila* strain AH-1 is able to degrade chitin due to extracellular chitinases, and *Flavobacterium* sp. acts as a cheater that uses chitin degradation products as nutrients (Jagmann et al., 2012). The biofilm formation of *Aeromonas* was enhanced when co-cultured with a *Bacillus cereus* strain that improves aggregation between bacteria (Cheng et al., 2014). In addition to mutualism, competitive behavior was shown between aquatic bacteria organized in biofilms. For instance, *A. hydrophila* exhibits antagonism against *L. pneumophila* through the production of bacteriocine-like substances. This interfering effect enhanced the detachment of *Legionella* from biofilms, contributing to its dissemination (Guerrieri et al., 2008).

The medicinal leech *Hirudo verbana* is the natural host of the two symbiotic bacterial species *A. veronii* and *Mucinovorans hirudinis* (Graf, 1999; Worthen et al., 2006; Nelson et al., 2015). Synergy occurs between the two bacterial endosymbionts because *A. veronii* associated with *M. hirudinis* forms larger mixed microcolonies than each species alone (Kikuchi and Graf, 2007). The mechanisms involved in this synergy are not yet known, and it is unknown whether it could be controlled by QS signaling and whether bacterial cooperation could initiate or influence leech gut colonization in the two symbionts.

## Quorum sensing systems in *Aeromonas*

Since its early description, intercellular communication via quorum signaling has gained a central place in bacterial sociobiology (Fuqua et al., 1994). Quorum sensing (QS) systems regulate a wide range of functions including bioluminescence, motility, extracellular virulence factors, and biofilm productions. Three different QS systems have been described in gram-negative bacteria, each composed of a sensor–autoinducer pair: type 1, type 2, and type 3 autoinducer systems.

### Type 1 autoinducer (AI-1) system

The AI-1 system was discovered within the LuxRI bioluminescent system in *Vibrio fisheri* (Engebrecht and Silverman, 1984) and is widespread among gram-negative bacteria including *P. aeruginosa* (Parsek et al., 1999). AI-1 signals are small molecules with a chemical structure based on N-acyl homoserine lactone (AHL), which is derived from common components of the bacterial metabolism, i.e., S-adenosyl methionine and acyl-acyl carrier proteins derived from fatty acid biosynthesis (Parsek et al., 1999). Depending on the species, the acyl chain length of AHLs varies from C4 to C18 and can be modified by unsaturation, methyl branches, and oxo- or hydroxyl substituents (Churchill and Chen, 2011).

#### AI-1 signaling in Aeromonas

**Figure 2:**
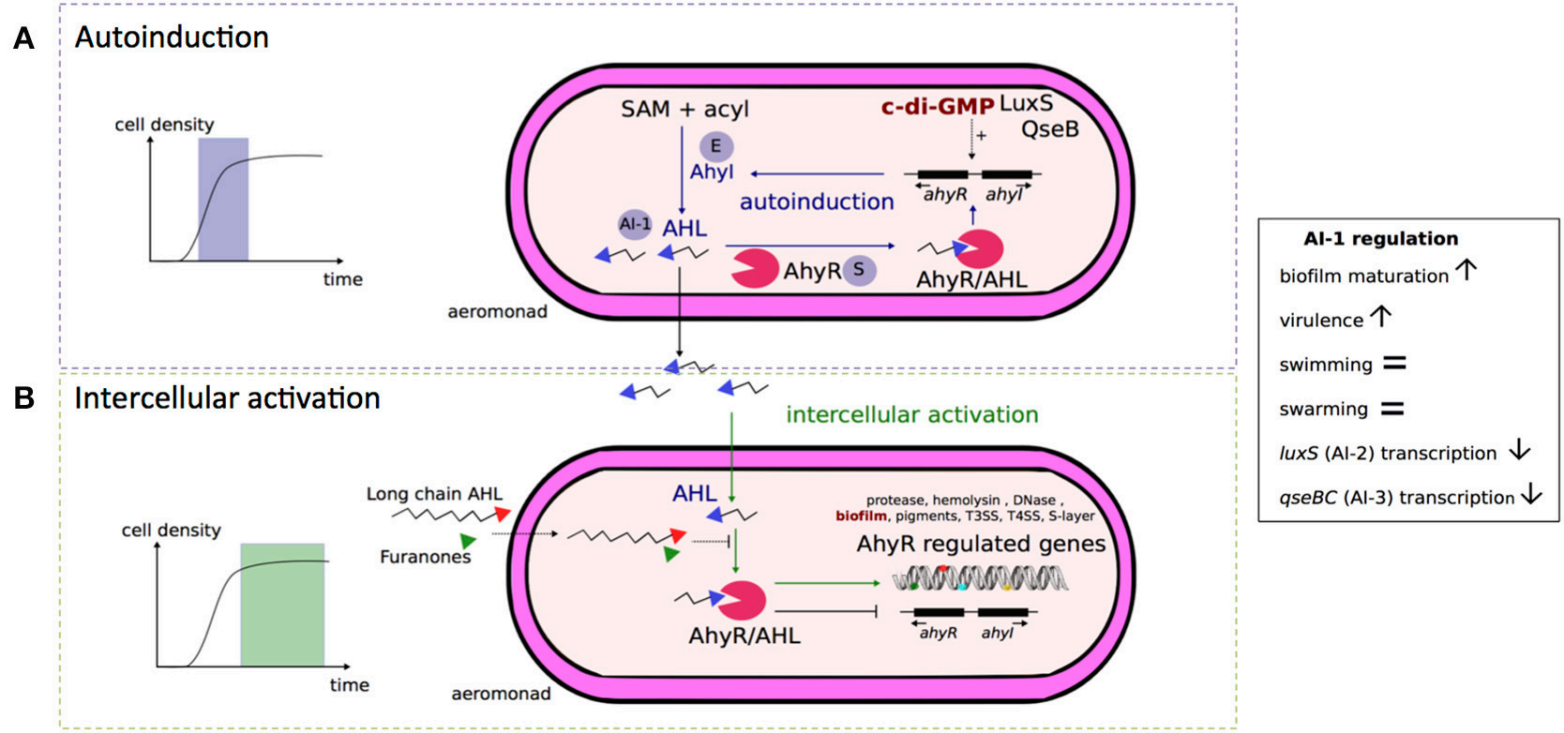
**Schematic representation of AI-1 quorum sensing system in ***Aeromonas*****. From an *in vitro* model of autoinducer 1 (AI-1) quorum sensing system of *A. hydrophila*, Garde et al. (2010) have distinguished two phases since the complex AI-1/receptor (AhyR) activates the quorum sensing loop of the initial AI-1 producer bacterial cell during exponential growth **(A)** or of other bacterial cells during the stationary phase **(B)** due to slow decay of the complex AI-1/receptor (AhyR). **(A)** Autoinduction occurs during exponential growth phase (Garde et al., 2010). In this phase, the enzyme (E) AhyI synthetizes AI-1 signal molecules of acyl-homoserine lactones (AHL) from S-adenosyl-methionine (SAM) and acyl–acyl carrier proteins (acyl) (Swift et al., 1997; Parsek et al., 1999). The protein AhyR is the sensor (S) of the AI-1 system and is activated by AHL molecules (Swift et al., 1997). Once activated, AhyR is a transcriptional regulator for the *ahyRI* locus encompassing AhyI and AhyR encoding genes, and participates in the auto-amplification loop (Kirke et al., 2004; Garde et al., 2010). The transcription of *ahyRI* locus is also likely enhanced (discontinuous traits) by the second messenger c-di-GMP and by AI-2 synthase LuxS or by AI-3 transcriptional regulatory protein QseB (Kozlova et al., 2012). The AHL molecules are freely diffusible across bacterial membranes and accumulate in the extracellular environment (Garde et al., 2010). **(B)** Intercellular activation occurs over an AHL concentration threshold corresponding to high cell density occurring at the stationary phase (Garde et al., 2010). Once activated by AHL molecules, AhyR is a transcriptional regulator for several genes associated to virulence and biofilm formation. In contrast to its action during the autoinduction phase, activated-AhyR negatively regulates the transcription of the *ahyRI* locus (Kirke et al., 2004). This AI-1 system is inhibited by exogenous long chain AHL or furanones (Swift et al., 1999; Ponnusamy et al., 2010) that may act as competitive inhibitors of AHL for AhyR binding. The AI-1 quorum sensing system negatively regulates the transcription of *luxS* and *qseBC* loci, encoding AI-2 synthase and AI-3 two components system, respectively (Kozlova et al., 2011, 2012).

A schematic model of the *Aeromonas* AI-1 quorum sensing system is presented in Figure 2. The LuxI-type enzyme, known as AhyI in *A. hydrophila* and AsaI in *A. salmonicida*, synthetizes AHL molecules (Figure 2A; Swift et al., 1997). AHLs interact with a cytoplasmic homolog of protein LuxR in *A. hydrophila* called AhyR (Figure 2), which is a transcriptional regulator of target genes, including the gene that encodes AhyI (Figure 2; Swift et al., 1997, 1999). During the exponential phase, AhyR and AhyI interplay in an activation loop of the AI-1 system. This leads to an auto-amplification effect called “autoinduction” (Figure 2A). AHLs freely diffuse across cellular membranes in and out of the *Aeromonas* cell and accumulate in the bacterial cell environment (Figure 2A). At the stationary phase over an exogenous AHL concentration threshold, the autoinduction phenomenon is suppressed while intercellular activation (i.e., “intercellular communication”) occurs between two bacterial cells and is the only active phenomenon (Figure 2B), as shown in *A. hydrophila* (Garde et al., 2010). The expression of AhyI is growth phase-dependent. Indeed, Kirke et al. (2004) showed that AhyI is produced during the exponential phase, but not during the post-exponential phase, when it is instead degraded. In the exponential phase, AhyR up-regulates the expression of AhyI and enhances AHL production (Figure 2A). In contrast, AhyR inhibits the expression of AhyI during the post-exponential phase (Figure 2B; Kirke et al., 2004). In addition, the transcription of *ahyRI* is enhanced by the expression of AI-2 synthase LuxS and the AI-3 response regulator QseB (Figure 2A; Kozlova et al., 2012).

AI-1 QS system may differ among bacterial genera, and may involve several kinds of LuxRI homologs. For example, *P. aeruginosa* harbors two distinct LuxRI homologs, LasRI and RhlRI (Lee and Zhang, 2015). In *Aeromonas*, only one AI-1 system has been described and is virtually present in every *Aeromonas* strain because LuxRI homologs were detected in all 73 tested strains, covering the known diversity in the genus *Aeromonas* (Jangid et al., 2007). The genes *ahyI* and *ahyR* from the locus *ahyRI* encode this system in *A. hydrophila* and are transcribed divergently, together with an intergenic region of 62 bp (Swift et al., 1997; Kirke et al., 2004). Homologs for these genes were also identified in other *Aeromonas* species (e.g., *A. salmonicida, Aeromonas molluscorum, A. veronii, A. media*, and *Aeromonas diversa*). A putative binding site for AhyR was identified in the intergenic region 10 bp upstream of the *ahyI* promoter (Kirke et al., 2004).

As shown in other gram-negative bacteria (e.g., *Agrobacterium, Erwinia*), the activation of AI-1 systems modulates fitness and virulence in *Aeromonas*, as reviewed in Table 2. Overall, AI-1 system activation in *Aeromonas* is associated with enhancement of biofilm maturation (without effects on swimming and swarming; Lynch et al., 2002; Khajanchi et al., 2009) and virulence. Indeed, the AhyRI system enhances the expression of numerous virulence factors in *Aeromonas*, including production of exoenzymes such as metalloproteases, serine proteases, hemolysin, amylase, DNAse, S-layer production, and pigment production (Table 2; Swift et al., 1999; Bi et al., 2007; Khajanchi et al., 2009; Schwenteit et al., 2011). Concerning the type III secretion system, the effect of AI-1 is unclear because of conflicting data. Some works have observed that T3SS is up-regulated by the AhyRI system, but other works observed no effect on the production and translocation of the T3SS effector AexU (Table 2; Khajanchi et al., 2009; Vilches et al., 2009). Type 1 QS also regulates the switch between two metabolic pathways. For instance, to avoid lethal acidification of the medium in the late growth phase, *Aeromonas* are able to switch from mixed acid fermentation to butanediol fermentation that produces fewer acid compounds (Table 2; Van Houdt et al., 2007).

**Table 2:** **Influences of quorum-sensing systems on the expression of virulence factors in ***Aeromonas*****.

**Virulence determinants**		Autoinducer system^*^	**Modulation effect**	**Strains**	**References**
Proteases	Metalloproteases, serine proteases	AI-1	Up-regulation	*A. hydrophila* AH-1N (WT, ΔahyI and ΔahyR)	Swift et al., 1999
	Proteases			*A. hydrophila* J-1 (WT and ΔahyR)	Bi et al., 2007
	AsaP1 protease			*A. salmonicida* subsp. *achromogenes* Keldur265-87 (WT and ΔasaI)	Schwenteit et al., 2011
	Metalloproteases			*A. hydrophila* {*A. dhakensis*} SSU (WT, ΔahyRI and ΔahyRI/ahyR+)	Khajanchi et al., 2009
Hemolysin	Aerolysin/Enterotoxin cytotoxic Act	AI-3	Up-regulation	*A. hydrophila* {*A. dhakensis*} SSU (WT and ΔqseB)	Khajanchi et al., 2012
	beta-hemolysin	AI-1	Down-regulation	*A. hydrophila* AH-1N (WT, ΔahyI and ΔahyR)	Swift et al., 1999
	hemolysin		Up-regulation	*A. hydrophila* J-1 (WT and ΔahyR)	Bi et al., 2007
Amylase		AI-1	Up-regulation	*A. hydrophila* J-1 (WT and ΔahyR)	Bi et al., 2007
DNase		AI-1	Up-regulation	*A. hydrophila* J-1 (WT and ΔahyR)	Bi et al., 2007
S-layer		AI-1	Up-regulation	*A. hydrophila* J-1 (WT and ΔahyR)	Bi et al., 2007
Brown pigment		AI-1	Up-regulation	*A. salmonicida* subsp. *achromogenes* Keldur265-87 (WT and ΔasaI)	Schwenteit et al., 2011
Type III secretion system		AI-1	Up-regulation (transcription)	*A. hydrophila* {*A. piscicola*} AH-3 (WT, ΔahyI and ΔahyR)	Vilches et al., 2009
			No effect (AexU production and translocation)	*A. hydrophila* {*A. dhakensis*} SSU (WT, ΔahyRI and ΔahyRI/ahyR+)	Khajanchi et al., 2009
Type VI secretion system		AI-1	Up-regulation	*A. hydrophila* {*A. dhakensis*} SSU (WT, ΔahyRI and ΔahyRI/ahyR+)	Khajanchi et al., 2009

* *The table described only AI-1 and AI-3 because the role of AI-2 in virulence factors expression is not yet demonstrated*.

#### Type of AHL produced

The different types of AHL produced by *Aeromonas* spp. strains, the methods of AHL identification and the origin of isolates are presented in Table 3. Culture supernatants of *A. hydrophila* AH-1 and *A. salmonicida* type strain NCIMB 1102^T^, as studied by bioassays and high-performance liquid chromatography (HPLC), showed that N-butanoyl homoserine lactone (C4-HSL), and N-hexanoyl homoserine lactone (C6-HSL) are the major autoinducers produced by aeromonads, and the ratio C4:C6 is 70:1 (Swift et al., 1997). In another study, all clinical tested strains of *A. hydrophila* (*n* = 20) and *Aeromonas sobria* (*n* = 2) produced either C4-HSL and C6-HSL or both, including 10 strains that produced an additional AHL, a putative N-pentanoyl homoserine lactone (C5-HSL; Chan et al., 2011). Other studies showed similar results, e.g., *A. caviae* YL12 (Table 3), although additional AHLs were not systematically characterized (Bruhn et al., 2005; Morgan-Sagastume et al., 2005; Medina-Martínez et al., 2006; Schwenteit et al., 2011; Chong et al., 2012; Huang et al., 2012; Chu et al., 2013; Ochiai et al., 2013; Lim et al., 2014; Zeng et al., 2014). The HPLC data confirmed results obtained with bioassays (Table 3). However, there were significant discrepancies in the AHL profiles between the HPLC and gas chromatography methods for *A. salmonicida* type strain (NCIMB1102^T^ = ATCC 33658^T^) because AHL with longer chains were detected only by gas chromatography (Swift et al., 1997; Cataldi et al., 2007). Compared to *Aeromonas, P. aeruginosa* produces two types of HSL, N-(3-oxododecanoyl)-HSL, and C4-HSL (Lee and Zhang, 2015).

**Table 3:** **Types of N-acyl homoserine lactone (AHL) produced by ***Aeromonas species*** from different isolation origins regarding the methods used for AHL analysis**.

**Strains**	**Origin of isolates**	**Methods of AHL identification and/or quantification**	**Types of produced AHL**	**References**
*A. hydrophila* AH-1	Natural isolate	TLC (CV026)/HPLC-HR-MS	C4++, C6	Swift et al., 1997
*A. salmonicida* NCIMB 1102^T^	Salmon	TLC (CV026)/HPLC-HR-MS	C4++, C6	
*Aeromonas* sp. (*n* = 3)	Activated sludge	TLC (CV026)/HPLC-HR-MS	C6, C4, or C6, C8, or C6, 3oxoC8	Morgan-Sagastume et al., 2005
*A. hydrophila* ATCC 7966^T^	Tin of milk with a fishy odor	TLC (pZLR4, CV026)/HPLC-HR-MS	C4	Bruhn et al., 2005
*A. hydrophila* 93-3-35, *A. salmonicida* 02-9-1	NR	TLC (pZLR4, CV026)/HPLC-HR-MS	C4, C6, UI	
*A. salmonicida* ATCC 33658^T^	Salmon	TLC (pZLR4, CV026)/HPLC-HR-MS	C4, C6, UI	
*Aeromonas* sp. (*n* = 13)	Food	TLC (CV026)	C4	Medina-Martínez et al., 2006
*A. hydrophila* ATCC 7966^T^	Tin of milk with a fishy odor	GC-MS	C8, C12, C14	Cataldi et al., 2007
*A. salmonicida* ATCC 33658^T^	Salmon	GC-MS	C8, C10, C12, C14	
*A. veronii* MTCC 3249	Mosquito midgut	GC-MS	C6, 3oxoC6, C7, 3oxoC7, C8, 3oxoC8, C9, 3oxoC9	Thiel et al., 2009
*A. hydrophila* (*n* = 4), *A. sobria* (*n* = 1)	Clinical sample	TLC (CV026)	C4	Chan et al., 2011
*A. hydrophila* (*n* = 6)	Clinical sample	TLC (CV026)	C4, C6	
*A. hydrophila* (*n* = 10)	Clinical sample	TLC (CV026)	C4, C6, putative C5	
*A. sobria* (*n* = 1)	Clinical sample	TLC (CV026)	C6, UI, UI	
*A. salmonicida* subsp. *achromogenes* Keldur 265-87	Diseased fish	TLC (pZLR4, CV026)/HPLC-HR-MS	C4	Schwenteit et al., 2011
*Aeromonas* GC1	Activated sludge	TLC (CV026)/NSI-MS, LC-ESI-FTMS	C4, C6, C8, C12	Chong et al., 2012
*A. veronii* MTCC 3249	Mosquito midgut	TLC (CV026)/HPLC-MS-NMR	6-carboxy-C6, C14	Jangid et al., 2012
*A. aquariorum* B2M05, *A. jandaei* B087, *A. salmonicida* B079	Urban river biofilm	TLC (CV026)	C4, UI	Huang et al., 2012
*A. hydrophila* B1M18, *A. media* Bill, *A. media* B026	Urban river biofilm	TLC (CV026)	C4, UI, UI	
*A. hydrophila* B015	Urban river biofilm	TLC (CV026)	C4, C6, UI	
*A. media* B1M53	Urban river biofilm	TLC (CV026)	C4, C8, 3oxoC8	
*A. veronii* B1M14	Urban river biofilm	TLC (CV026)	3oxoC8, UI	
*Aeromonas* sp.	Activated sludge	TLC (CV026)	C4, C6	Ochiai et al., 2013
*A. hydrophila* (*n* = 24)	Fresh water, diseased fish	TLC (CV026)	C4, C6	Chu et al., 2013
*A. caviae* YL12	Compost	TLC (CV026)/HPLC-HR-MS	C4++, C6	Lim et al., 2014
*A. hydrophila* S1_073	Rhizosphere of Wetland Plants	TLC (CV026)	C4, C6, U	Zeng et al., 2014
*A. media* S1_063	Rhizosphere of Wetland Plants	TLC (CV026)	C4, UI, UI	
*A. aquariorum* S2_004	Rhizosphere of Wetland Plants	TLC (CV026)	C4, UI	
*A. sobria AS7*	Spoiled turbot	Parallel streaks (CV026, A136)/GC-MS	C4, C6, C8++, C10++, C12	Li et al., 2016

*AHL, N-acyl homoserine lactone; HPLC-HR-MS, high performance liquid chromatography/high resolution mass spectrometry; GC-MS, Gas chromatography-mass spectrometry; HPLC-MS-NMR, high performance liquid chromatography/mass spectrometry- nuclear magnetic resonance; NSI-MS, nanospray ionization- mass spectrometry; LC-ESI-FTMS, liquid chromatography/electrospray ionization-Fourier transform mass spectrometry; TLC, thin layer chromatography; CV026, reporter strain Chromobacterium violaceum CV026; pZLR4, reporter strain Agrobacterium tumefaciens NTL4(pzLR4); A136, reporter strain Agrobacterium tumefaciens A136; NR, not reported; UI, unidentified; ++, major AHLs produced*.

#### AI-1 inhibitors

The *Aeromonas* AI-1 system is inhibited *in vitro* by exogenous AHLs harboring long chains, i.e., 10–14 carbons HSL, leading to decreased production of exoproteases (Swift et al., 1999). Consistently, the 3-oxo-C10-HSL produced by *Vibrio anguillarum* inhibits protease activities from *A. salmonicida* and *A. hydrophila* (Rasch et al., 2007). Similarly, the synthetic 2(5H)-furanone derived from the competitive inhibitor of AHL produced by the marine algae *Delisea pulchra*, exhibited QS inhibition activity against C4-HSL and C6-HSL, molecules usually produced by aeromonads (Ponnusamy et al., 2010).

### Type 2 autoinducer (AI-2) system

**Figure 3:**
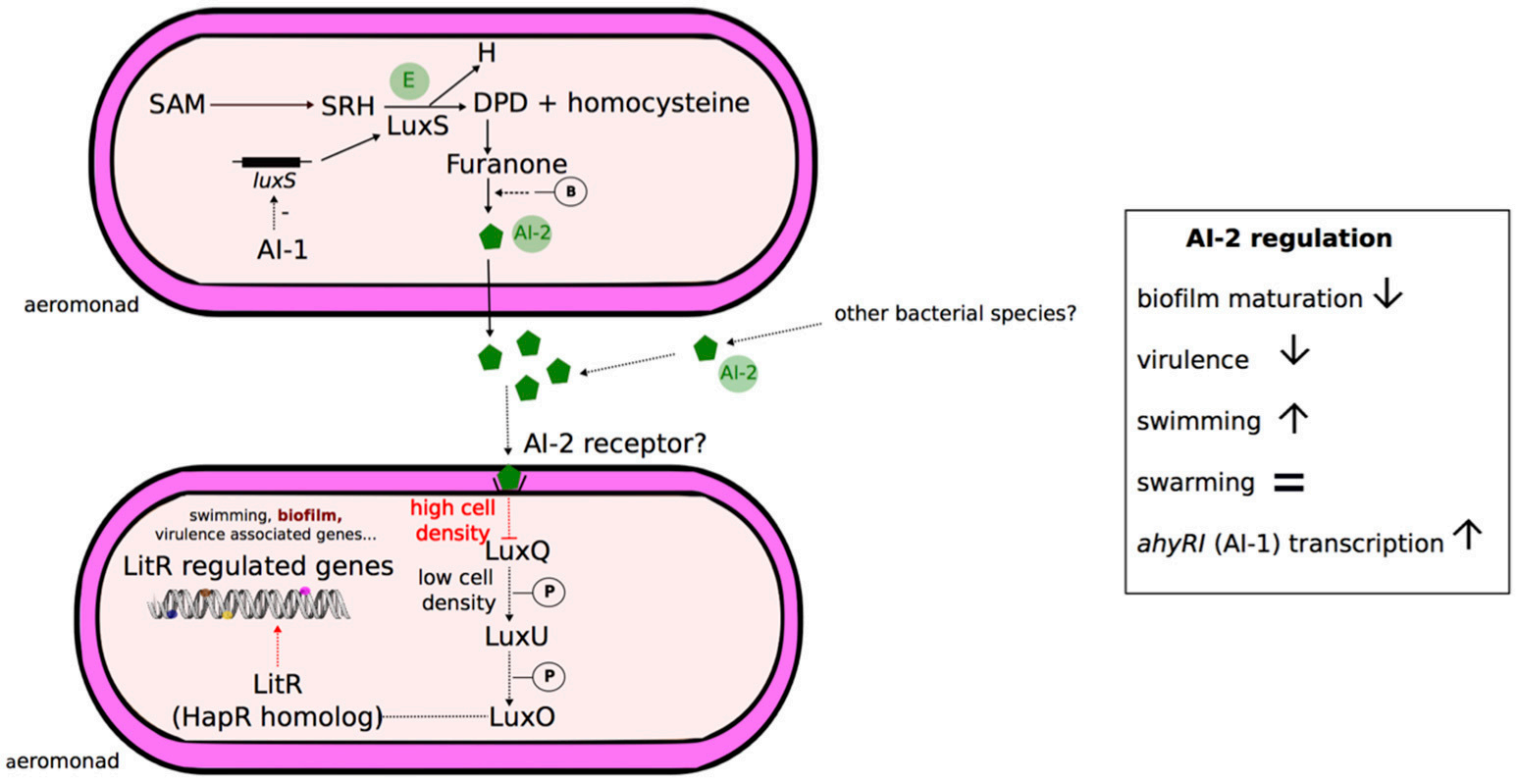
**Schematic representation of AI-2 quorum sensing system in ***Aeromonas*****. Aeromonads are able to produce the AI-2 synthase enzyme (E) LuxS, and AI-2 (autoinducer 2) quorum sensing system has been detected in the genus (Kozlova et al., 2008). In the bacterial AI-2 quorum sensing systems, LuxS catalyzes the cleavage of S-ribosyl-homocysteine (SRH) derived from S-adenosyl-methionine (SAM) in homocysteine and 4,5-dihydroxy-2,3-pentanedione (DPD) (Xavier and Bassler, 2003). DPD spontaneously cyclizes to form a furanone, which can possibly react with borate (-B) depending on the bacterial species (discontinuous traits), and leading to AI-2 molecule formation (Chen et al., 2002; Miller et al., 2004). Based on studies in *Vibrio*, it has been shown that in absence of AI-2, LuxQ generates a phosphorylation cascade (-P) via LuxU and ultimately LuxO. LuxO is the response regulator that represses the master regulatory protein HapR (*V. cholerae*). At high cell density, AI-2 freely diffusible molecules reach a threshold and bind the LuxP periplasmic receptors. The autoinducer signal is transduced by the LuxP/AI-2 complex, inactivating the transmembrane sensor kinase LuxQ and subsequently leading to LuxO inactivation, which lifts repression of HapR and influences gene expression (Bassler et al., 1994; Henke and Bassler, 2004). However, the AI-2-internalization step of aeromonads is not yet known (discontinuous traits) and no luxP homolog were detected into their genomes (Kozlova et al., 2008). Signal transduction may involve the proteins LuxQ, LuxU, LuxO and subsequently the transcriptional regulator LitR (homolog of HapR), but the level of proof is so far only genetic (Kozlova et al., 2011). Overall, the AI-2 activation system in *Aeromonas* is associated with inhibition of biofilm maturation, enhancement of swimming and a decrease in virulence (Kozlova et al., 2008). The transcription of *luxS* locus is likely inhibited (discontinuous traits) by AI-1 quorum sensing system (Kozlova et al., 2011).

The AI-2 QS system was initially described in *Vibrio harveyi* to control the expression of bioluminescence in response to fluctuation of bacterial population density (Bassler et al., 1994; Surette et al., 1999). A putative schematic model of *Aeromonas* AI-2 quorum sensing system is presented in Figure 3. AI-2 molecules are produced and detected by many gram-positive and gram-negative bacteria, including *Aeromonas* spp. and are considered a “universal signal autoinducer” with functions in interspecies cell-to-cell communication (Figure 3; Fong et al., 2001; Miller et al., 2004; Federle, 2009). AI-2 molecules are by-products of S-adenosyl-methionine (as AI-1) and correspond to a furanosyl borate diester in *V. harveyi* (Chen et al., 2002) or a variant lacking borate in *Salmonella enterica* (Figure 3; Miller et al., 2004). These molecules are synthesized by the enzyme LuxS (Xavier and Bassler, 2003) and freely diffuse across bacterial membranes (Figure 3). Under conditions of high cell density in *V. cholerae*, AI-2 molecules bind to a periplasmic receptor and lead indirectly to the derepression of the transcriptional regulator HapR (Figure 3; Henke and Bassler, 2004; Xavier and Bassler, 2005).

The genomes of *A. hydrophila* ATCC 7966^T^ and *A. hydrophila* {*A. dhakensis*} SSU contain homologs for AI-2 synthase LuxS and enzymes involved in signal transformation (AI-2 sensor kinase/phosphatase LuxQ, phosphorelay protein LuxU, regulatory protein LuxO) and a LitR encoding-gene, a homolog for the transcriptional regulator HapR of *V. cholerae*, and Lit-R-regulated genes (Figure 3; Kozlova et al., 2011). The transcription of *luxS* is negatively regulated by the expression of the locus *ahyRI* (Kozlova et al., 2011). The functions of AI-2 in the *A. hydrophila* {*A. dhakensis*} strain SSU have been studied by constructing Δ*luxS* deletion mutants. AI-2 is involved in the up-regulation of swimming motility and the down-regulation of biofilm formation and bacterial virulence in a murine model (Kozlova et al., 2008).

### Type 3 autoinducer (AI-3) system

**Figure 4:**
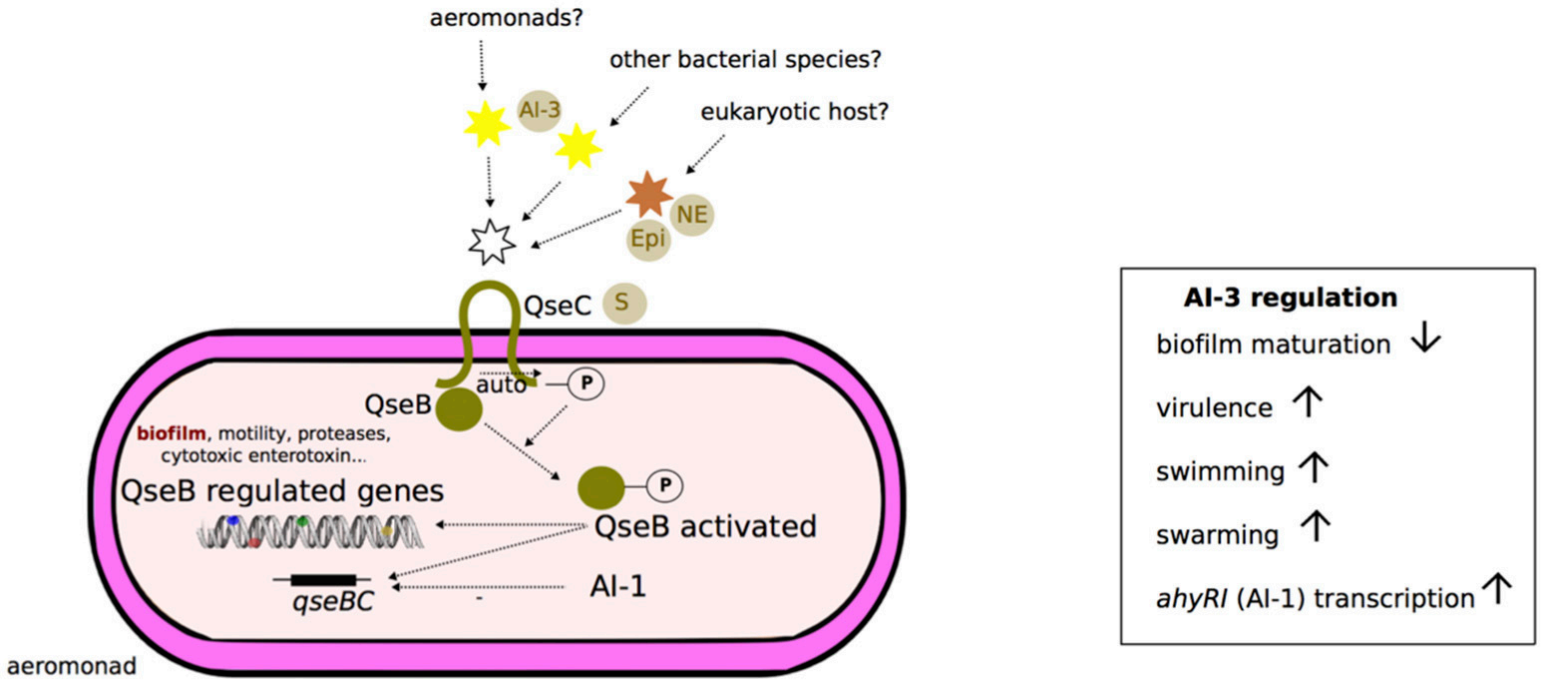
**Schematic representation of AI-3 quorum sensing system in ***Aeromonas*****. Although, the two-component system QseB/QseC was characterized in *Aeromonas* (Khajanchi et al., 2012), the synthesis of autoinducer 3 (AI-3) signals is not yet known in this genus (discontinuous traits). According to the *Escherichia coli* model, the transmembrane protein QseC is a sensor (S) that can bind at its periplasmic domain: (i) signal molecules of AI-3 from other cells of a bacterial clone or from other bacterial species, or (ii) catecholamines (epinephrine, Epi, or norepinephrine, NE) from a eukaryotic host (Sperandio et al., 2002; Clarke et al., 2006). QseC then undergoes autophosphorylation (-P) at its cytoplasmic domain. The signal is then transmitted by phosphorylation (-P) to the transcriptional regulatory protein QseB. Subsequently, activated QseB-P binds to the transcription regulator domains of virulence-associated genes (flagella, shiga-like toxin, type III secretion system components, and effectors) and autoregulates its own operon *qseBC* (Sperandio et al., 2002; Clarke et al., 2006). Overall, the AI-3 system activation in *Aeromonas* is associated with inhibition of biofilm maturation, enhancement of swimming and swarming and an increase in virulence (Khajanchi et al., 2012). The transcription of *qseBC* locus is likely inhibited (discontinuous traits) by AI-1 quorum sensing system (Kozlova et al., 2012).

The type 3 autoinducer (AI-3) system is a hormone-like signal transduced by the two-component QseBC system in which QseC is the sensor kinase and QseB the response regulator (Figure 4). A putative schematic model of the *Aeromonas* AI-3 quorum sensing system is presented in Figure 4. AI-3 is suspected to behave similar to eukaryotic hormones because QseC is also a bacterial adrenergic receptor for the eukaryotic host hormones epinephrine and norepinephrine and is thus involved in interkingdom cross-signaling (Figure 4; Sperandio et al., 2003). AI-3 molecules are usually produced by gastrointestinal microbiota, e.g., in the context of symbiotic relationships between microbiota and host (Clarke et al., 2006). The periplasmic sensing domain of QseC is conserved among several gram-negative bacterial species (e.g., *E. coli, S. enterica*; Clarke et al., 2006).

Khajanchi et al. (2012) have identified and characterized the QseBC QS system in *A. dhakensis* that encodes a functional homolog for *E. coli* QseBC. In *Aeromonas*, AI-3 enhances swarming and swimming motility and virulence (e.g., hemolytic activity, protease and cytotoxic enterotoxin production, toxicity in murine models) and negative regulation of biofilm formation, as shown in the Δ*qseB* deleted mutant of the *A. hydrophila* {*A. dhakensis*} strain SSU (Khajanchi et al., 2012). The transcription of *qseB* and *qseC* genes is negatively regulated by the AI-1 QS system (Kozlova et al., 2012; Figure 4).

### How are biofilm and quorum sensing interconnected in *Aeromonas?*

Bacteria commonly form dense communities in biofilms (Nadell et al., 2009). Consequently, biofilm lifestyle has emerged as a suitable model to study cell-to-cell signaling mechanisms. The three QS systems described in *Aeromonas* spp. are able to coordinate and influence biofilm formation, maintenance, and possibly dispersion. In addition, the major intracellular second messenger c-di-GMP has an impact on biofilm formation of *Aeromonas* spp. through QS systems, which is similar to the role observed in *P. aeruginosa* (Harmsen et al., 2010).

### AI-1 signaling positively regulates *Aeromonas* biofilm maturation

After the initial attachment phase, the AHL synthase AhyI and AHLs (C4-HSL and C6-HSL) are produced within the biofilm during microcolony formation and biofilm maturation (Lynch et al., 2002), as observed in other gram-negative bacteria and in particuliar *P. aeruginosa* (Davies et al., 1998; Huber et al., 2001; Labbate et al., 2004). Evidence of AI-1 effects on biofilms in aeromonads is provided by mutagenesis experiments and the effect of specific inhibitors.

Mutagenesis experiments clearly demonstrated that AI-1 influence depends on the biofilm development stage. The early attachment phase was not altered in Δ*ahyI* (AI-1 synthase) or Δ*ahyR* (AI-1 sensor/regulator) mutants of the *A. hydrophila* strain AH-1N (Lynch et al., 2002). Consistent with this, both swimming and swarming motilities were able to influence biofilm formation and were conserved in a double Δ*ahyRI* mutant of *A. hydrophila* {*A. dhakensis*} strain SSU (Khajanchi et al., 2009). However, the later phases of biofilm maturation were affected by AI-1; the AH-1N Δ*ahyI* (AI-1 synthase) mutant developed a biofilm less differentiated than that of the wild type (WT; Lynch et al., 2002). In addition, the number of viable cells was reduced in Δ*ahyI* mutant biofilms compared to WT, but there was no difference in the viability of planktonic cells between Δ*ahyI* and WT (Lynch et al., 2002). These defects in biofilm formation were partially restored by addition of exogenous AI-1, C4-HSL (Lynch et al., 2002). Confirming these observations, an *A. hydrophila* {*A. dhakensis*} SSU Δ*ahyRI* mutant was defective and unable to form a well-structured biofilm, with alterations in filamentation, strain aggregation, and the (Khajanchi et al., 2009). In contrast to the Δ*ahyI* (AI-1 synthase) mutant, the Δ*ahyR* (AI-1 sensor/regulator) mutant of *A. hydrophila* strain AH-1N retains the ability to form mature biofilm, suggesting that the role of AHLs in the formation of large mushroom-like microcolonies does not depend on AhyR (Lynch et al., 2002) but presumably depends on transcriptional regulators other than AhyR. For instance, Kozlova et al. (2011) reported the presence of two loci that encode AhyR homologs in the strain *A. hydrophila* {*A. dhakensis*} SSU. In summary, these mutagenesis experiments showed that AI-1 molecules and AI-1 synthase enhanced biofilm formation.

Evidence for the role of AI-1 on biofilm formation is reinforced by studies focusing on AI-1 inhibitors, i.e., compounds that bear AI-1 quorum-quenching properties. For instance, chestnut honey decreases the level of C4-HSL molecules produced by the *A. hydrophila* strain CECT 839^T^ and leads to significant inhibition of biofilm formation (Truchado et al., 2009). Synthetic 2(5H)-furanone, derived from natural furanones produced by the marine algae *D. pulchra*, inhibits *in vitro* AHLs, including those usually produced by aeromonads (C4-HSL and C6-HSL), and results in biofilm inhibition in a strain of *A. hydrophila* (Ponnusamy et al., 2010). Vanillin exhibited quorum-quenching activity against aeromonad AHLs and reduced *A. hydrophila* biofilm by altering its architecture and decreasing surface cell-density and protein content (Ponnusamy et al., 2013). *Mentha piperata* essential oil and menthol have a quorum-quenching effect against C6-HSL. Sub-minimal inhibitory concentrations of mint essential oil and menthol decrease extracellular polymeric substance production and biofilm formation in the *A. hydrophila* strain WAF-28 (Husain et al., 2015). Examples of inhibition on biofilm formation displayed by several AI-1 inhibitors confirm dependence of biofilm formation to AI-1 QS.

### AI-2 and AI-3 operate in negative feedback regulation on *Aeromonas* biofilm formation

Overall, the planktonic form is promoted by AI-2 and AI-3 because these two QS systems operate in negative feedback regulation in the biofilm formation of *Aeromonas* (Kozlova et al., 2008, 2012; Khajanchi et al., 2012).

A Δ*luxS* mutant of *A. hydrophila* {*A. dhakensis*} strain SSU defective in AI-2 showed enhanced amount of biofilm (Kozlova et al., 2008). Meanwhile, swimming motility, was reduced when *luxS* was deleted (Kozlova et al., 2008), suggesting that LuxS up-regulates swimming motility. This result should not be viewed as conflicting because it favors a switch to a planktonic lifestyle and is compatible with down-regulation of biofilm formation exhibited by the AI-2 QS system. Indeed, polar flagella are involved in biofilm formation at the initial stage, but this appendage is still fully expressed in the planktonic life of aeromonads (Canals et al., 2006). AI-3 signaling seems to exert similar effects to AI-2 on the down-regulation of biofilm formation and up-regulation of swimming motility as observed in the strain *A. hydrophila* {*A. dhakensis*} SSU and also up-regulates swarming motility (Khajanchi et al., 2012; Kozlova et al., 2012).

### Role of c-di-GMP on *Aeromonas* biofilm maturation

Cyclic di-guanosine monophosphate (c-di-GMP) is a secondary messenger that is mainly involved in the transition from a planktonic to sessile lifestyle in the domain *Bacteria* and was well-studied in *P. aeruginosa* or *V. cholerae* (Simm et al., 2004; Tischler and Camilli, 2005; Kuchma et al., 2007). For a comprehensive review, see Römling et al. (2013). Synthesis and degradation of c-di-GMP are under control of peptide domains widespread and universally conserved in bacteria: GGDEF (di-guanylate synthase activity) and EAL (phosphodiesterase activity), respectively. The *A. hydrophila* ATCC 7966^T^ genome contains 32 proteins with GGDEF, 9 proteins with EAL and 13 proteins with both (Rahman et al., 2007). In *Aeromonas*, the overexpression of the GGDEF domain enhances biofilm formation and inhibits swimming (polar flagellum dependent) and swarming motility (lateral flagella dependent) whereas, overexpression of the EAL domain leads to opposite effects. Thus, it is thought that c-di-GMP positively regulates biofilm maturation (sessile life) and negatively affects bacterial motility for aeromonads (planktonic life; Rahman et al., 2007; Kozlova et al., 2008, 2011, 2012; Khajanchi et al., 2012).

AI-2 and AI-3 QS are involved in the control of biofilm by c-di-GMP. For instance, one gene coding for a GGDEF domain protein, namely *AHA0701*, is located immediately downstream of the LuxS encoding-gene (AI-2 synthase gene) in the genomes of *A. hydrophila* ATCC 7966^T^ and *A. hydrophila* {*A. dhakensis*} SSU strains (Seshadri et al., 2006; Kozlova et al., 2011). Overexpression of *AHA0701* increased the number of surface-attached cells and enhanced biofilm formation (Kozlova et al., 2011) and putatively led to extensive EPS production (Kozlova et al., 2012) by down-regulation of AI-2 synthase transcription. Corroborating the involvement of AI-2 and AI-3 QS in the c-di-GMP dependent regulation of sessile lifestyle in *Aeromonas*, the phenotype of strains that overproduced GGDEF domains varied in the same direction as Δ*luxS* (AI-2) and Δ*qseB* (AI-3) mutants vs. WT, i.e., increasing the maturation of biofilm and decreasing bacterial motility (Kozlova et al., 2008, 2011, 2012; Khajanchi et al., 2012). The regulation of sessile life through c-di-GMP also involves the AI-1 system in *Aeromonas*, as proved by the increase in C4-HSL production and enhancement of *ahyI* and *ahyR* transcription when c-di-GMP increases. In conditions of c-di-GMP overproduction, the transcript levels of *luxS, qseB*, and *qseC* genes also increased, but this influence is dependent on the AI-1 system (Kozlova et al., 2011). Finally, the *ahyRI* locus is required to promote c-di-GMP-mediated biofilm maturation (Rahman et al., 2007; Kozlova et al., 2011). Moreover, several homologs for genes encoding effectors of c-di-GMP signaling involved in biofilm formation of *V. cholerae* or *P. aeruginosa* have been found in *Aeromonas* spp. genomes. Among them, two transcriptional regulators containing the DNA-binding domains VpsR/FleQ and VpsT/CsgAB, and one master regulator for biofilm formation FleN were identified (Kozlova et al., 2011). In *A. dhakensis*, the transcription of effectors VpsR/FleQ, VpsT/CsgAB, and FleN is enhanced by c-di-GMP and is also modulated by AI-1, AI-2, and AI-3 systems, with opposite effects (Kozlova et al., 2011, 2012).

In summary, there is cross-talk between the three QS systems AI-1, AI-2, and AI-3 in the c-di-GMP regulation of the sessile life in *Aeromonas* (Kozlova et al., 2011, 2012).

### Further research

A large amount of knowledge is available for understanding biofilm formation, QS systems, and interconnections between biofilms and QS in aeromonads. However, some knowledge is still missing for a comprehensive understanding of aeromonad social life. The type 1 autoinducer (AI-1) system is well-described, but further investigations are needed to specify the AHL profiles actually produced according to species, growth phase and origin of isolates. The AI-2 and AI-3 QS systems are poorly described and deserve to be more deeply investigated. Although, the AI-2 system has proved to be involved in *Aeromonas* biology thanks to mutagenesis evidence, the system could be better characterized. More specifically, homologs for the AI-2 receptor (LuxP in *V. harveyi*) have not been detected in the genome of *A. hydrophila* {*A. dhakensis*} strain SSU or *A. hydrophila* ATCC 7966^T^ (Kozlova et al., 2011), and the chemical nature of AI-2 produced from aeromonads should be clarified. Further studies are also needed to explore the AI-3 system and characterize the AI-3 signal(s) at the level of bacterial clone and within host interacting microbiota. Further studies designed to better understand this regulation system in *Aeromonas* spp. in high-density cell and biofilm situations are needed. Above all, QS and biofilm are the beginning of the understanding of complex and fine systems involved in aeromonad social life. Once the Pandora's box opened, additional exciting, unstudied questions will arise. Mixed biofilm structures and lifestyles likely result from a balance between cooperation and competition that we will have to unravel. Changes in gene expression during different stages of biofilms are another field to explore (Nadell et al., 2009). In addition, packed communities secrete into the local environment and share beneficial metabolites that are costly to produce, with benefits for individuals (Dumas and Kümmerli, 2012). These “public goods” may be exploited by non-producing mutants, i.e., cheaters (Heilmann et al., 2015). How biofilm structure, population dynamics within biofilm is impacted by cheaters is unknown. Understanding these aspects will help us to better understand the social life of aeromonads.

## Fields impacted by the multi-cellular behavior of *Aeromonas*

### Flocs, biofilms, and aeromonads in activated sludge

Activated sludge is a typical process for treating sewage and industrial wastewater taking advantage of the sociobiology of aeromonads, i.e., biofilm formation, QS and synergistic behavior. Members of the genus *Aeromonas* have been estimated to account for ~2% of the total biomass of activated sludge (Kampfer et al., 1996). Aeromonads organized in biofilm and aggregates have the biotechnological interest in the removal of organic pollutants (Chong et al., 2012), nitrogen (Chen et al., 2014), and phosphorus (Andersson et al., 2011) and are resistant to extreme conditions of pH, salinity, heavy metals, and temperature (Chen et al., 2014; Mohd Yasin et al., 2014).

Flocs in activated sludge are microbial aggregates enchased in a matrix of EPS composed of carbohydrates, proteins, lipids, and nucleic acids. These polymers come from bacterial cell metabolism, autolysis, and exogenous wastewater particles (Frølund et al., 1996). An analogy between biofilms and flocs is generally drawn because these bioaggregates correspond to a microbial surface producing EPS that improve the cohesion of the supracellular structure. The ability to flocculate was shown *in vitro* with a strain of *Aeromonas* sp. isolated from activated sludge (Chong et al., 2012).

To reduce hydraulic retention times, biofilm techniques are increasingly used to increase the surface of biodegradation. In a model of mixed biofilms composed of strains regularly present in activated sludge, Andersson et al. (2011) reported that mixed species biofilms that included *A. hydrophila* were associated with synergistic effects on denitrification and phosphorus removal. A shift in the composition of the produced EPS was also highlighted, with a significant rise in the carbohydrate fraction in the *Brachymonas denitrificans* model mixed with *A. hydrophila*, compared with single species biofilms. New polysaccharides were also detected but only from a mixed culture of *A. hydrophila* and *Comamonas denitrificans* or *Acinetobacter calcoaceticus* (Andersson et al., 2011). Another multi-species biofilm containing *Comamonas testosteroni* and *A. hydrophila* strains display synergistic biofilm formation, enabling better resistance of the biofilm to the repetitive physical and chemical shocks occurring in wastewater (Li et al., 2008). These interactive adaptive behaviors within the biofilm have great potential to improve wastewater treatment efficiency.

From typical activated sludge flocs containing *Alpha*-, *Beta*-, and *Gammaproteobacteria*, as well as members of *Bacteroidetes* and *Actinobacteria*, Li and Zhu (2014) showed by a quorum quenching method that AI-1 QS is necessary for the aerobic granulation of flocs and qualitatively and quantitatively regulates the content of exopolymeric substances. *Aeromonas* sp. in activated sludge are reported to produce AHLs (Chong et al., 2012; Ochiai et al., 2013). Interestingly, AHLs were localized in flocs community but absent from the aqueous phase (Chong et al., 2012). Moreover, the exogenous addition of AHLs led to increase the chitinase activity of the aeromonads isolated from activated sludge and of the whole activated sludge (Chong et al., 2012). Furthermore, QS signaling within activated sludge can also be modulated by bacteria degrading AHLs, such as *Acinetobacter* (Ochiai et al., 2013). All these data support that *Aeromonas* in flocs display QS-dependent social behavior improving water treatment.

### Role in virulence

Biofilm formation is involved in the pathogenesis of numerous human colonizations and/or infections (e.g., healthcare-associated infections) and persistent infections (e.g., chronic infection during cystic fibrosis, endocarditis, biliary tract infections, periodontitis, otitis media, wounds; Lebeaux et al., 2013). Aeromonads are occasionally involved in biofilm-associated colonization of medical devices, leading to infection such as lenses-associated keratitis (Willcox et al., 2001; Pinna et al., 2004) and central venous catheter-associated bloodstream infection (Andreoli-Pinto and Graziano, 1999; Tang et al., 2014). These reports suggest that biofilm acts as a virulence or patho-adaptive factor in *Aeromonas* infections. Consequently, biofilms are constantly evaluated in virulence studies. However, the correlation between biofilm producer and aeromonad pathogenesis is still weak. Presumably, the ability to produce biofilm *in vitro* may explain long-term digestive epithelium colonization by some strains whether they are associated with an infection (Janda and Abbott, 2010). Bfp type IV pili and polar/lateral flagella were suspected to be factors of intestinal colonization (Kirov et al., 1999, 2004). However, the production of lateral flagella is unlikely to be compulsory in digestive colonization because it is inconstantly expressed in *Aeromonas* spp. clinical isolates recovered from diarrheal feces (Kirov et al., 2002). The level of evidence for the exact contribution of biofilm to *Aeromonas* pathogenicity needs to be improved, and further studies are welcomed.

Because it was shown that several virulence determinants (e.g., alpha-hemolysin, cholesterol acetyltransferase, lipase, and serine protease) were overproduced at high cell density in late exponential/stationary phase, cell density could be a prerequisite for aeromonad pathogenic behavior and virulence expression (MacIntyre and Buckley, 1978; Whitby et al., 1992; Anguita et al., 1993). This suggests a pivotal role for QS in the course of infection. Both AI-1 and AI-3 signaling enhance the expression of some virulence determinants of aeromonads (Table 2; Figures 2, 4) and increase lethality in a murine model, as proved by the virulence-reduced phenotypes exhibited by Δ*ahyRI* and Δ*qseB* mutants vs. WT (Khajanchi et al., 2009, 2012). In contrast, AI-2 signaling is associated with a down-regulation of bacterial virulence in a murine model (Kozlova et al., 2008), although molecular targets have yet to be identified. The pathogenesis of *Aeromonas* infections remain to be better understood, but QS-based cross-talk is indubitably involved in the expression of virulence from a threshold of cell density.

As described in *P. aeruginosa*, AHL molecules (e.g., 3-oxo-C12-HSL) are not only involved in bacterial virulence regulation but also interact with several eukaryotic cells and play a role in the immuno-modulation of the host response (Liu et al., 2015). Khajanchi et al. (2011) have reported a similar immune-modulatory effect with C4-HSL, C6-HSL, and N-3-oxo-C12-HSL in septicemic mice infected with *A. hydrophila* {*A. dhakensis*}. Indeed, AHL-based pretreatment was associated with reduced levels of cytokines/chemokines in tissues, increased neutrophil recruitment from blood (C6-HSL), enhanced bacterial clearance, limitation of clinical symptoms, and increase in survivability for infected mice. In addition, the AHL-based pretreatment increased *in vitro* bacterial phagocytosis by murine macrophages. Thus, it seems that exogenous AHLs act as a signal for the activation of host response to infection (Khajanchi et al., 2011). Both promising QS inhibitors that interfere with aeromonad QS, e.g., menthol (Husain et al., 2015), and some purified QS molecules (Khajanchi et al., 2011), represent new therapeutic perspectives to fight against aeromonosis threatening multi-drug antibiotic resistance.

There is increasing evidence that suggests the influence of multicellular microbial interaction during the course of infection, in the case of mixed infections driven by cooperation and/or competition (Trejo-Hernández et al., 2014; Armbruster et al., 2016). Microbial interactions may be particularly critical in the *Aeromonas* genus. Indeed, infections caused by *Aeromonas* are frequently polymicrobial, i.e., caused by *Aeromonas* and other bacterial genera (30–80% of the cases), (Lamy et al., 2009; Lay et al., 2010; Figueras and Beaz-Hidalgo, 2015); aeromonads are mainly associated with enterobacteria, *Staphylococcus aureus* and anaerobes. In 5–10% of human aeromonosis, infections are caused by heterogeneous populations associating several clones of *Aeromonas* spp. (Lamy et al., 2009). For some pairs of aeromonads co-isolated from clinical samples, the virulence during infection in *Caenorhabditis elegans* was higher for pairs than for each individual strain (Mosser et al., 2015). The exact mechanism of pathogenicity and aeromonads interactions during mixed infections remains unsolved, but intercellular communication likely plays a critical role in cooperation and/or competition behaviors. Ponnusamy et al. (2016) have also reported complex cross-talk in which two different clones of *A. hydrophila* affect each other, leading to a changing course of infection in a murine model. Indeed, an antagonistic effect through direct and host-mediated elimination was shown. In parallel, authors have observed a synergistic effect on the dissemination via local tissue barrier damage (Ponnusamy et al., 2016).

## Conclusion

Aeromonads display diverse multicellular behaviors that represent many strategies to grow and persist in natural and anthropized environments. These adaptive behaviors potentially influence the virulence of *Aeromonas*. Living in biofilm provides aeromonads with a high cell-density that enhances interaction between bacteria through QS systems. The three QS systems play an important regulatory role in the expression of a wide range of functions for aeromonads including biofilm formation, motility, and virulence, and these systems harbor differences in their regulative influences. The AI-1 system enhances sessile life by promoting biofilm while AI-2 and AI-3 enhance planktonic life by inhibiting biofilm maturation and increasing bacterial motility. These effects on planktonic and sessile life are also influenced by the second messenger c-di-GMP. Considerable interconnections exist between the three QS systems and the biofilm formation in the genus *Aeromonas*, as in other bacterial species. Efforts should be made to develop models in order to obtain accurate and robust data to better understand multicellular aspects of *Aeromonas* biology. In human infections, the role of *Aeromonas* biofilm in human gastro-intestinal colonization and the influence of bacterial clones in aeromonad-containing mixed infections deserve to be studied to better decipher aeromonosis patho-physiology.

## Author contributions

Conceived and designed the work: ET, EJ, BL; Performed survey and drafted the paper: ET; Critically revised the manuscript: EJ, BL. All authors read and approved the final manuscript.

### Conflict of interest statement

The authors declare that the research was conducted in the absence of any commercial or financial relationships that could be construed as a potential conflict of interest.
